# Becoming a “Hungry Mind”: Stability and Change in Need for Cognition across Adolescence

**DOI:** 10.3390/jintelligence12100103

**Published:** 2024-10-15

**Authors:** Jeroen Lavrijsen, Evelien Aerts, Franzis Preckel, Alicia Ramos, Karine Verschueren

**Affiliations:** 1 Department of Mathematics, KU Leuven, 3000 Leuven, Belgium; 2 School Psychology and Development in Context, KU Leuven, 3000 Leuven, Belgium; evelien.aerts@kuleuven.be (E.A.); alicia.ramos@kuleuven.be (A.R.); karine.verschueren@kuleuven.be (K.V.); 3 Department of Psychology, Trier University, D-54286 Trier, Germany; preckel@uni-trier.de

**Keywords:** need for cognition, stability, home environment, cognitive stimulation, developmental trajectory, cognitive ability

## Abstract

Need for Cognition has been established as a key intellectual investment trait shaping students’ academic development. However, little is yet known about its malleability, particularly in youth. This study investigated stability and change in Need for Cognition in a large longitudinal sample of 3409 adolescents from 166 classes in 27 schools in Flanders. Participants reported on their Need for Cognition in Grades 7, 8, 11 and 12. First, the latent rank-order stability of Need for Cognition between Grades 7 and 11 was found to be *r* = 0.50. This stability was of a similar magnitude to that of the Big Five personality traits observed in the same sample and larger than that of academic intrinsic motivation. Second, trajectories of Need for Cognition over time were found to vary between individuals. In particular, three trajectory classes could be identified, differentiated mainly by the initial level of Need for Cognition (i.e., a low, medium, and high trajectory). Finally, cognitive ability, cognitive stimulation at home, and parental autonomy support, but not parental educational level, were associated with higher odds of belonging to the high Need for Cognition trajectory and lower odds of belonging to the low Need for Cognition trajectory.

## 1. Introduction

Need for Cognition refers to an individual’s tendency to seek out, engage in, and enjoy effortful cognitive activity (Cacioppo and Petty 1982). As such, Need for Cognition is considered to be a key intellectual investment trait, that is, a personality trait that guides how, when, and where individuals apply and invest their intellectual abilities (Ackerman 1996). Previous research has demonstrated that Need for Cognition predicts students’ school engagement (Lavrijsen et al. 2021; Luong et al. 2017) and academic performance (Liu and Nesbit 2023). To support students’ academic outcomes, it may thus be important to understand if and how Need for Cognition can be promoted in youth.

To date, however, little is yet known about the malleability of Need for Cognition. As an investment trait, Need for Cognition was originally conceptualized as a relatively stable personality characteristic (Cacioppo and Petty 1982). However, such a conceptualization does not imply complete rigidity; rather, personality traits appear to be to some degree malleable, particularly in youth (Bleidorn et al. 2022; Borghuis et al. 2017), with personality change possible through recurrent deviations (personality ‘states’) from one’s usual trait level (‘personality trait’) (Wrzus and Roberts 2017). Specifically for Need for Cognition, Cacioppo et al. (1996) already suggested that it could be fostered by letting children repeatedly experience that cognitive activities are rewarding and enjoyable; such recurring experiences were then hypothesized to crystallize over time into a more endurable, positive disposition towards cognitive effort. However, to date, the malleability of Need for Cognition across adolescence has received only limited research attention.

The present study investigated the stability of and change in Need for Cognition across adolescence. In a large longitudinal sample of adolescents, who were followed between Grades 7 and 12, this study contrasted the rank-order stability of Need for Cognition with that of established personality and motivational constructs. In addition, it addressed potential interindividual variability in the intraindividual developmental trajectories in Need for Cognition across adolescence. Finally, this study investigated which individual and environmental factors explained differences in Need for Cognition trajectories between adolescents.

### 1.1. Rank-Order Stability of Need for Cognition

In general, the degree of malleability of a certain construct can be examined in terms of its rank-order stability over time. Rank-order stability indicates how consistently individuals maintain their relative standing among their age group on a measure over two different points in time; it is usually quantified as the correlation coefficient between two measurements. Overall, the rank-order stability of personality constructs depends on both the age group of the respondents and the time lag between measurements (Bleidorn et al. 2022).

To the best of our knowledge, no study has yet targeted the rank-order stability of Need for Cognition across adolescence. However, two recent longitudinal studies yielded indications of the rank-order stability of Need for Cognition in either younger or older youth. A first study among elementary school children (M_age_ = 8.40 at the first measurement point) over a one-year interval reported the rank-order stability of Need for Cognition to equal *r* = 0.56 (Bergold and Steinmayr 2023). A second study among late adolescents (M_age_ = 16.43 at the first measurement point) over a time interval of 2.5 years found the rank-order stability in Need for Cognition to be *r =* 0.59 (Bergold et al. 2023).

To situate the degree of malleability of Need for Cognition, the rank-order stability of Need for Cognition can be compared to that of established personality traits, such as the Big Five traits. Rank-order stabilities of the Big Five traits have already been extensively documented. A recent meta-analysis summarizing 189 studies reported an average rank-order stability of *r* = 0.61 across the Big Five traits (Bleidorn et al. 2022), with a slightly higher rank-order stability for openness and extraversion and a slightly lower stability for conscientiousness and neuroticism. The meta-analysis further showed trait stability to increase throughout adolescence and to decrease with an increasing time lag between measures. These findings allow us to estimate the rank-order stability of Big Five traits for a specific age group and for a specific time lag between measurements. For the set-up of the present study (i.e., with the Big Five traits measured between Grades 7 and 11), the meta-analysis would project an expected average rank-order stability of *r* = 0.52.

In the present study, the rank-order stability of Need for Cognition was compared not only with the Big Five traits but also with academic intrinsic motivation. Although Need for Cognition is conceptualized as a personality trait, it also encompasses a clear motivational element, as it reflects an individual’s intrinsic desire to engage in cognitive activities. Particularly in youth, Need for Cognition might share conceptual overlap with academic intrinsic motivation, that is, the extent to which children study out of interest and enjoyment. Granted, Need for Cognition relates to cognitive effort in general, irrespective of the nature or setting; it is thus not intrinsically tied to the school context. However, in youth, many opportunities for thinking are presented at school. Hence, students who like thinking can be expected to better enjoy most school tasks; in contrast, students who like their school work may adopt a more positive attitude towards cognitive activities in general (Lavrijsen et al. 2021). Indeed, empirically, strong correlations (i.e., around *r* = 0.50) between Need for Cognition and academic motivation have been observed (Gottfried et al. 2017; Lavrijsen et al. 2023). When investigating the malleability of Need for Cognition, this potential link between Need for Cognition and academic motivation is important because academic motivation is often considered to be more malleable than personality traits. Indeed, motivational frameworks such as self-determination theory (Ryan and Deci 2000) emphasize that academic motivation is highly dependent on the learning environment, for example, because it is associated with teachers’ use of need-supportive instructional strategies (Stroet et al. 2013). A recent study of the same age group as the present study (M_age_ = 12.75 years) estimated the rank-order stability of academic intrinsic motivation for mathematics to equal *r* = 0.38 over a time lag of 1.5 years (Held and Hascher 2023), which is indeed below corresponding estimates for the Big Five traits. Hence, to situate the malleability of Need for Cognition, this study contrasted the rank-order stability to that of the Big Five traits and academic motivation. To date, no study has compared the rank-order stability of these constructs within a single longitudinal sample.

### 1.2. Interindividual Variability in the Development of Need for Cognition

Beyond rank-order stability, which captures the stability of the relative standings of individuals, this study also considered the interindividual variability in the intraindividual development of Need for Cognition over time, that is, between-person differences in within-person change (Gordon 2009). Examining this interindividual variability adds to a more comprehensive perspective on the stability of and change in a construct (Curran and Bauer 2011). Indeed, whereas low rank-order stability implies a relatively high degree of interindividual variability in trajectories (as this variability is needed to explain the observed changes in relative standings), a high rank-order stability does not need to imply a low variability in individual trajectories. For example, when all individuals with high initial values have an increase over time and all individuals with low initial values have a decrease, this would preserve relative standings and thus yield a high rank-order stability. Still, under such circumstances, individual trajectories would clearly differ between individuals (i.e., some increasing, some decreasing) and thus imply a relatively large degree of interindividual variability in intraindividual change.

This interindividual variability in developmental trajectories can be statistically analyzed with growth curve models (Curran et al. 2010). In such models, individual trajectories are modeled to result from (a) a fixed effect describing the overall mean trajectory and (b) random components describing the deviation of individual trajectories from this mean, both in terms of initial levels (i.e., a random intercept) and trend over time (i.e., a random slope). Furthermore, distinct subgroups (classes) in the population of individual trajectories can then be identified with techniques such as latent class growth analysis and growth mixture modeling (Jung and Wickrama 2008).

Given that the available evidence suggests a moderate rank-order stability of Need for Cognition, it can be expected that individuals develop, to some extent, differently over time. The two longitudinal studies on the development of Need for Cognition in elementary school children (Bergold and Steinmayr 2023) and late adolescents (Bergold et al. 2023) both yielded a statistically significant random time slope in Need for Cognition, suggesting substantial interindividual variability in the intraindividual trajectories over time. Yet, more research is needed to map interindividual variability in trajectories throughout adolescence.

### 1.3. Individual and Environmental Factors Affecting the Development of Need for Cognition

Finally, this study addressed which individual and environmental factors may explain individual differences in initial levels of Need for Cognition and in changes over time. Understanding why adolescents adopt a higher Need for Cognition than others is crucial from both a research and an applied perspective, as it would allow us to tune these factors to maximally promote the development of Need for Cognition in youth. In the literature, Need for Cognition has been suggested to relate both to the cognitive ability of the individual and to environmental factors, particularly in the home environment (Cacioppo et al. 1996).

#### 1.3.1. Cognitive Ability

On the individual level, several researchers have suggested that the development of Need for Cognition is related to individuals’ cognitive abilities. Theoretically, intellectual investment theory posits that high cognitive ability would increase the probability of encountering success in cognitive activities (environmental success hypothesis), which would make children better enjoy such efforts and increase their self-efficacy towards cognitive tasks (Ackerman 1996; Ziegler et al. 2012). A stronger confidence in their ability to successfully complete cognitive tasks would make children more likely to seek out and persist in cognitive effort and thus boost their Need for Cognition (Jebb et al. 2016). On the other hand, a high Need for Cognition may incentivize individuals to seek out and invest in cognitively challenging learning activities, which would further enhance their cognitive abilities (environmental enrichment hypothesis). In sum, intellectual investment theory suggests a reciprocal positive relationship between Need for Cognition and cognitive ability (Ziegler et al. 2012).

Empirically, cross-sectional research has found cognitive ability and Need for Cognition to be weakly to moderately positively correlated (Fleischhauer et al. 2010; Hill et al. 2013; Lavrijsen et al. 2023). Moreover, the aforementioned longitudinal study among elementary school children found that mathematical ability predicted increases in Need for Cognition over time, although the observed increases were small (Bergold and Steinmayr 2023). However, the aforementioned study among late adolescents found that whereas cognitive ability was related to the initial level of Need for Cognition, cognitive ability did not predict changes in Need for Cognition over time (Bergold et al. 2023). In a recent experimental study (Ackermann et al. 2022), a training intervention improving cognitive abilities in preschoolers and elementary school children led to a small increase in Need for Cognition shortly after training (Cohen’s *d* for the pretest–post-test difference equal to 0.21). However, in a follow-up study three months after the training, the positive training effect on Need for Cognition had vanished. In sum, whereas cognitive ability and Need for Cognition seem to be weakly to moderately correlated, evidence for the effects of cognitive ability on changes in Need for Cognition over time is inconclusive.

#### 1.3.2. Home Environment

In addition to cognitive ability, factors in the home environments of children may affect the development of Need for Cognition in children and youth. Theoretically, Need for Cognition has been postulated to be nurtured when parents regularly provide their children with cognitive activities that these children experience as enjoyable and rewarding, as this may instill a positive disposition towards cognitive effort in children (see Aerts et al. 2024 for this issue). The benefits of such a cognitively stimulating home environment have already been observed with regard to academic intrinsic motivation. For example, in a longitudinal study mapping the development of academic intrinsic motivation from childhood into early adolescence, cognitive stimulation positively predicted later academic intrinsic motivation (Gottfried et al. 1998). Similarly, frequently exposing children to new experiences and novel ideas has been found to positively affect academic intrinsic motivation in early adolescents (Gottfried et al. 2016). Given these findings for academic motivation, one could expect cognitive stimulation at home to also positively affect the development of Need for Cognition.

To date, no empirical study has related cognitive stimulation at home directly to Need for Cognition. However, a number of studies have followed a similar line of thought to propose using the socio-economic statuses of families to predict Need for Cognition, as socio-economic status tends to correlate with home exposure to cognitive activities (Rosen et al. 2020). Possibly surprisingly, most studies typically reported Need for Cognition to be only weakly related to socio-economic background (Colling et al. 2022; Padgett et al. 2010; Steinmayr et al. 2023). Among elementary school children, longitudinal research showed that the parental educational level was unrelated to changes in Need for Cognition over time, although the number of books at home predicted small increases in Need for Cognition (Bergold and Steinmayr 2023). Similarly, among late adolescents, parental occupational status was found to be unrelated to changes in Need for Cognition over time (Bergold et al. 2023).

Arguably, it might be not just the extent to which parents provide cognitive activities to their children that is decisive for children’s Need for Cognition, but also how they do so (Aerts et al. 2024). In particular, when parents force participation in cognitive activities on their children (e.g., hothousing; Leslie 2014), this might possibly backfire. Indeed, in such conditions, children would not learn to associate cognitive effort with enjoyment and appreciation but rather with pressure and demands. In contrast, an autonomy-supportive parenting style, which would acknowledge the perspective of the child, could be argued to be more suited to improving Need for Cognition in children. The benefits of autonomy-supportive parenting have been repeatedly demonstrated in research on academic intrinsic motivation. For example, a meta-analysis summarizing 36 studies found that higher parental autonomy support was related to higher academic intrinsic motivation in children and adolescents (Vasquez et al. 2016). This line of reasoning might suggest that Need for Cognition could be fostered as well when parents acknowledge their children’s autonomy in cognitive home activities.

### 1.4. Present Study

The present study investigated the stability of and change in Need for Cognition in a longitudinal sample of adolescents.

First, the rank-order stability of Need for Cognition between Grades 7 and 11 was compared to that of the Big Five personality traits and to that of academic intrinsic motivation. Given that Need for Cognition was conceptualized as a personality trait but may have some conceptual overlap with academic intrinsic motivation, we expected a medium rank-order stability for Need for Cognition, somewhere in between that of the Big Five traits and academic intrinsic motivation.

Second, longitudinal growth curves in Need for Cognition across adolescence were estimated and interindividual variability in these trajectories was assessed. Based on previous research, we expected to find a significant random time slope in Need for Cognition over time. We then explored whether distinct subgroups of trajectories over time could be identified using latent class growth modeling.

Finally, we considered whether adolescents’ cognitive ability and a set of home environment variables (i.e., parental educational level, cognitive stimulation at home, and parental autonomy support) would predict membership of Need for Cognition trajectory classes. We tentatively expected cognitive ability, cognitive stimulation at home, and parental autonomy support to predict membership of classes with higher Need for Cognition; regarding parental educational level, we expected small associations at best.

## 2. Materials and Methods

### 2.1. Sample and Procedure

Data were drawn from the longitudinal talent-study (e.g., Lavrijsen et al. 2021). This study was initiated in 2017 and followed a large community sample of 3409 early adolescents from 166 classes in 27 Flemish schools (49.6% boys, M_age_ = 12.41 years, age range 8.85–14.33 years, SD_age_ = 0.49) across secondary education. Schools were recruited for the study via open calls for participation within school networks. Among participating schools, all students who had successfully completed primary school (known as the A-stream) were invited to participate. This study was approved by the institutional ethical committee, and consent for participation was obtained from adolescents, parents, and teachers. Students had a slightly more advantaged social background than the Flemish secondary education student population, with 21.2% of the sample receiving financial governmental support for low-income families (compared with 25.7% of the general population), 11.9% speaking a different language at home (16.9% of the general population), and 14.1% having a mother without a secondary school degree (18.0% of the general population).

This study consisted of six waves (Grade 7 (Fall and Spring), Grade 8 (Fall and Spring), Grade 11 (Fall), Grade 12 (Spring)). Initially, schools registered to participate in the first four waves; accordingly, all schools continued their participation throughout Grades 7 and 8 (i.e., *N*_G7_ = 27 and *N*_G8_ = 27). However, when the last two waves were added to the study, 10 schools decided not to further participate in Grade 11 (i.e., *N*_G11_ = 17) and an additional 6 decided not to participate in Grade 12 (i.e., *N*_G12_ = 11). In addition, a number of students left the participating schools over the course of secondary education. For these reasons, the number of participants was reduced over the course of this study (*n*_G7_ = 3.409; *n*_G8_ = 2.861; *n*_G11_ = 1.567; *n*_G12_ = 988). Dropout was significantly related to adolescents’ Need for Cognition, with small differences in average levels between those continuing their participation and those dropping out (i.e., the average initial Need for Cognition among those who participated in all waves equaled 3.17, whereas the average initial Need for Cognition among those dropping out in Grade 8 equaled 2.84 (*d* = −0.41), among those dropping out in Grade 11 2.96 (*d* = −0.26) and among those dropping out in Grade 12 3.17 (*d* = −0.00)). Further analyses showed that average initial Need for Cognition in Grade 7 was somewhat higher in schools that continued participation throughout this study (average value 3.05) compared to schools discontinuing their participation (average value 2.93, *d* = −0.13).

Finally, in Grade 7 (Fall), parents were asked to complete at home a survey capturing parental educational level, cognitive stimulation at home, and parental autonomy support. For 2.886 students (84.7%), at least one parent completed the survey.

### 2.2. Measures

All survey measures were rated on a Likert-scale ranging from 1 to 5, with the endpoints labeled “Does not apply to me” and “Fully applies to me”. The reliabilities of measures were compared with threshold values by DeVellis (1991), which suggests that alpha’s above 0.70 indicate good reliability for research purposes (with alpha = 0.65 to be the threshold for “minimal acceptability”).

Adolescents’ Need for Cognition was assessed in Grades 7 (Spring), 8 (Spring), 11 (Fall), and 12 (Spring). It was measured with a Dutch translation of the German 14-item Need for Cognition scale by Preckel and Strobel (2017), for which the psychometric properties and validity for use with children and adolescents are well documented (Keller et al. 2016; Preckel and Strobel 2017). In the present study, the measure demonstrated very high reliability (α_G7_ = 0.92/α_G8_ = 0.93/α_G11_ = 0.92/α_G12_ = 0.93)^1^.

The Big Five personality traits were assessed in Grade 7 (Fall) and Grade 11 with the Quick Big Five (Vermulst and Gerris 2005). Each of the five traits was assessed with 6 items. Overall, the reliabilities of each trait measure were good, with the exception of Openness in Grade 7, which was only minimally acceptable (Openness α_G7_ = 0.68/α_G11_ = 0.84; Conscientiousness α_G7_ = 0.76/α_G11_ = 0.80; Extraversion α_G7_ = 0.84/α_G11_ = 0.72; Agreeableness α_G7_ = 0.89/α_G11_ = 0.90; Neuroticism α_G7_ = 0.87/α_G11_ = 0.87).

Academic intrinsic motivation was assessed in Grade 7 (Fall) and Grade 11 with 4 items from the subscale of the Academic Self-Regulation Questionnaire by Ryan and Connell (1989). The subscale exhibited good internal consistency (α_G7_ = 0.87/α_G11_ = 0.86).

Parental educational level was surveyed in Grade 7 (Fall) by asking parents to report the highest qualification level achieved. This was coded as 0 = no secondary education; 1 = secondary education; 2 = Bachelor’s; and 3 = Master’s or beyond. As is common in research on social background (Avvisati 2020), the highest educational level among both parents was used to indicate parental educational level.

Cognitive stimulation at home was measured in the parent questionnaire in Grade 7 (Fall) with 5 items from a scale by Stevens et al. (2014). These items assessed different forms of cognitive stimulation at home (i.e., buying or borrowing books for the child, watching documentaries together, discussing the news with the child, attending an exhibition together, discussing school). The internal consistency of the scale was minimally acceptable (α_G7_ = 0.66). If both parents responded, their responses were averaged.

Parental autonomy support (e.g., I let my child plan his/her schoolwork himself/herself) was surveyed among parents in Grade 7 (Fall) with 4 items from the scale by Cheung et al. (2016). Internal consistency of the scale was good (α_G7_ = 0.77). If both parents responded, their responses were averaged.

Finally, cognitive ability test was measured in Grade 7 (Fall) with a well-validated Flemish test (CoVaT-CHC) that assessed both fluid and crystallized intelligence (Magez et al. 2015). An IQ-score for each adolescent was calculated based on a comparison of test results with a norming sample.

### 2.3. Analyses

All analyses were conducted with Mplus 8 (Muthén and Muthén 2017).

First, we analyzed the rank-order stability of Need for Cognition, the Big Five personality traits, and academic intrinsic motivation between Grades 7 and 11. As random measurement error can affect stability estimations, we modeled Need for Cognition, the Big Five traits and academic intrinsic motivation as latent variables. Given the complexity of the measurement model (i.e., three different latent constructs measured at two time points, with Need for Cognition in particular indicated by a relatively high number of items), we decided to parcel items (i.e., we averaged subsets of items to indicate each construct). Indeed, it has been argued that such parceling alleviates a number of psychometric problems as compared to individual items, and it in particular improves modeling efficiency and stabilizes parameter estimates (Matsunaga 2008). In our measurement model, each latent construct at each measurement point was thus indicated by three parcels (i.e., for Need for Cognition, parcels consisted of items 1–5, 6–10, and 11–14; for the Big Five traits, parcels consisted of items 1–2, 3–4, and 5–6; and for academic intrinsic motivation, parcels consisted of items 1–2, 3, and 4).

We first tested the invariance of this measurement model over time, that is, we tested whether model fit of the measurement model would be affected by imposing equality constraints for corresponding factor loadings and intercepts of these parcels between Grades 7 and 11 measurements (e.g., we imposed an equality between the factor loading of Need for Cognition on the first parcel in Grade 7 and the corresponding factor loading in Grade 11). In particular, we tested both metric invariance (i.e., invariance in the equality of factor loadings) and scalar invariance (i.e., invariance of factor intercepts). As χ2 is known to be highly sensitive to sample size (Putnick and Bornstein 2016), we compared model fit of measurement models using three indicators of model fit, that is, the Comparative Fit Index (CFI), the Root Mean Square Error of Approximation (RMSEA), and the Standardized Root Mean Squared Residual (SRMR). As a rule of thumb, invariance then could be established when the decrease in CFI was not larger than 0.010 (Cheung and Rensvold 2002), when increases in RMSEA were not larger than 0.010, and when increases in SRMR were not larger than 0.025, respectively (Chen 2007).

After establishing measurement invariance, we then estimated latent intercorrelations between Need for Cognition, the Big Five traits, and academic intrinsic motivation between the Grade 7 and 11 assessments.

Second, to study longitudinal growth in Need for Cognition across adolescence, a series of univariate latent growth curve models were specified in which individual Need for Cognition levels in Grades 7, 8, 11 and 12 were explained by an intercept, a linear slope, and a quadratic slope term after establishing scalar measurement invariance over time for these measures. In the growth curve models, loadings were set to take unequal spacings between measurements into account (e.g., for the linear term, *t* = 0 for Grade 7, *t* = 1 for Grade 8, *t* = 4 for Grade 11, and *t* = 5 for Grade 12). For each variable, a model with only an intercept term was compared with a model with both an intercept and a linear term, and then to a model with an intercept, a linear, and a quadratic term. The Bayesian Information Criterion (BIC) was used to see which of these models yielded the best fit with the data (lower BIC values indicate better model fit).

Using the best fitting models from the previous step of the analysis, a series of latent class growth models (Jung and Wickrama 2008) were specified to identify distinct classes in trajectories of Need for Cognition over time. Several criteria were used to decide on the optimal number of latent classes. First, BIC was evaluated between solutions with different classes. As BIC generally tends to decrease with each additional class, we considered at which number of classes the decrease in BIC elbowed. Second, the quality of the classification was addressed by estimating the entropy of the solution, which measures the accuracy of assigning individuals to trajectory classes. Third, the qualitative substantive value of each class was examined: when a solution with *k* classes included two classes that exhibited only minor substantive differences in between, the more parsimonious *k* − 1 class solution was preferred. Finally, solutions yielding very small classes (e.g., only 5% of the sample) were not preferred.

In a final set of analyses, class membership was related to cognitive ability, parental educational level, cognitive stimulation at home, and parental autonomy support. Class membership was predicted by using the three-step Bolck–Croon–Hagenaars (BCH) method (Bolck et al. 2004). The BCH method yields for each individual and each class a weight, which reflects the assignment probability of the individual to that class. However, in this study, the BCG method sometimes yielded negative weights, which inhibited further estimations. Hence, instead of predicting weights, we decided to predict for each individual the most likely class (i.e., the class with the highest positive weight) by cognitive ability, parental educational level, cognitive stimulation at home, and parental autonomy support using a multinomial multivariate regression model.

## 3. Results

### 3.1. Descriptive Statistics

First, Table 1 presents the descriptive statistics of all variables in this study. For reasons of space, for variables assessed more than once, only the first measurement occasion was used; correlations for the other measurement occasions can be found in the Appendix A. Among the individual and environmental predictors, Need for Cognition was positively related to cognitive ability, cognitive stimulation at home, parental educational level, and parental autonomy support. Across all four measurement points, intercorrelations between Need for Cognition measures were between 0.38 and 0.60 (see Appendix A).

### 3.2. Measurement Invariance

Table 2a reports the model fit indicators for testing metric invariance (i.e., invariance in the equality of factor loadings) and scalar invariance (i.e., invariance of factor intercepts) of Need for Cognition, the Big Five traits, and academic intrinsic motivation between Grades 7 and 11. Table 2a shows that when testing metric invariance, all changes in fit indices were within the limits required to establish invariance (i.e., ΔCFI > −0.010, ΔRMSEA < 0.010, ΔSRMR < 0.025). When testing scalar invariance, only the decrease in CFI (−0.011) slightly surpassed this threshold; all other comparisons supported scalar invariance, and the model still yielded a satisfactory model fit.

Table 2b reports the model fit indicators for the testing metric and scalar invariance of Need for Cognition over all measurement points. Regarding metric invariance, all changes in fit indices were within the limits required to establish invariance. Regarding scalar invariance, only the increase in RMSEA (+0.013) slightly surpassed the threshold (ΔRMSEA < 0.010), whereas all other comparisons supported scalar invariance, and the scalar model still yielded a satisfactory model fit. In sum, these results thus allowed for further analyzing these variables over time.

### 3.3. Latent Correlations

Next, latent intercorrelations of Need for Cognition, the Big Five traits, and academic intrinsic motivation between Grade 7 and Grade 11 assessments were estimated. Table 3 shows that the rank-order stability of Need for Cognition was estimated at *r* = 0.50. This appeared to be in the same order of magnitude as the rank-order stability of the Big Five traits, which averaged *r* = 0.51 over all five traits, although with some heterogeneity between traits (in particular, higher stability for openness and lower stability for neuroticism). In contrast, the rank-order stability of academic intrinsic motivation was *r* = 0.38 and thus clearly below that of Need for Cognition.

### 3.4. Latent Growth Curves

Table 4 shows that a latent growth curve model including a linear growth factor yielded a lower BIC than (i.e., should be preferred over) an intercept-only model, with a modest decrease in Need for Cognition over time. In addition, in the linear growth model, there was statistically significant interindividual variability both around the intercept and around the linear term, suggesting interindividual variability in trajectories in Need for Cognition over time.

Next, adding a quadratic growth factor was observed to further decrease BIC, also yielding a very good overall model fit (CFI = 0.992; SRMR = 0.024; RMSEA = 0.057). Moreover, the estimate for the quadratic growth factor was found to be statistically significant. For these reasons, we decided to build on the quadratic model to further determine trajectory classes. The positive estimate for the quadratic term suggested that Need for Cognition followed a U-curve over time. In this model, interindividual variability around the linear growth factor was now marginally significant, whereas variability around the quadratic term was not statistically significant.

### 3.5. Trajectory Classes

To determine the trajectory classes of Need for Cognition, Table 5 presents an overview of the selection criteria described in the “Analysis” section for the one- to five-class solutions. First, the reduction in BIC was large when a second and a third class were added, but it became much less pronounced when adding a fourth or fifth class. The entropy values peaked at the three-class solution. Moreover, the three-class solution still yielded three reasonable large classes (i.e., containing 61.4%, 18.3%, and 20.3% of the adolescents, respectively), whereas the four-class solution included a class with only 5.6% of the adolescents. The additional class in the four-class solution also added little substantive value to the interpretation of Need for Cognition trajectories for the more parsimonious three-class solution (i.e., it just split up the high Need for Cognition trajectory into two highly similar subsets). For these reasons, the three-class solution was chosen as the best-fitting solution.

**Figure 1:**
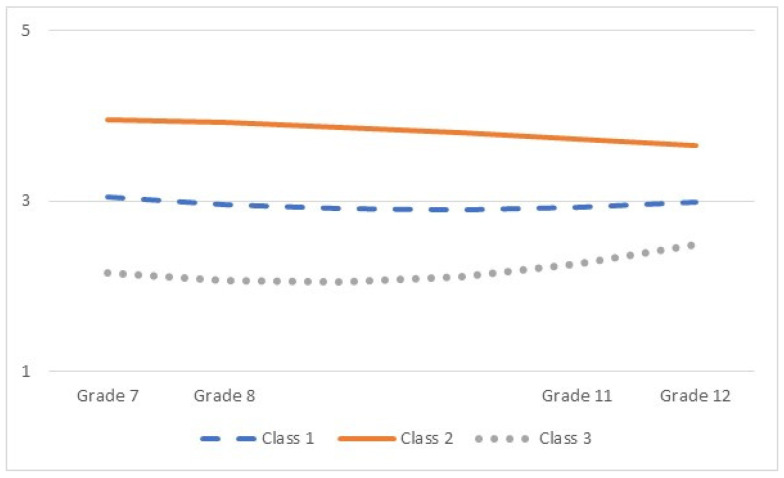
A graphical display of the three trajectory classes of Need for Cognition.

For the three-class solution, Table 6 provides estimates of intercepts and linear and quadratic slopes. To facilitate the interpretation of the quadratic trajectories, Figure 1 depicts trajectories per class. The three classes were differentiated mainly by their initial level of Need for Cognition. A majority of adolescents belonged to Class 1 (“Medium Need for Cognition”), with a medium initial level of Need for Cognition and a small negative linear but positive quadratic trajectory over time, culminating in a quite stable level over time (see Figure 1). In Class 2, adolescents reported a high initial level of Need for Cognition (“High Need for Cognition”). Whereas the linear and quadratic slope growth factors were not significant, they were both negative in this class; graphically, this added up to a small decline over time (Figure 1). Finally, adolescents in Class 3 reported a low initial level in Need for Cognition and, similar to Class 1, a negative linear and positive quadratic trajectory over time. Graphically, this class reported an uptick in Need for Cognition towards the end of secondary education, although levels remained relatively low.

### 3.6. Predicting Class Membership

Finally, the probability of belonging to either the high or low Need for Cognition trajectory class, relative to the medium class, was predicted by a multinomial multivariate regression model. Table 7 shows that cognitive ability (moderate effect), cognitive stimulation at home (weak effect), and parental autonomy support (weak effect) positively predicted belonging to the high Need for Cognition class. Furthermore, cognitive ability (moderate effect) and cognitive stimulation at home (weak effect) negatively predicted belonging to the low Need for Cognition class. Parental educational level was not found to significantly affect class membership probabilities, beyond the other predictors.

## 4. Discussion

The present study investigated the stability of and change in Need for Cognition in a large longitudinal sample of adolescents, who were followed between Grades 7 and 12.

### 4.1. Rank-Order Stability of Need for Cognition Compared to Motivation and Personality

First, this study contrasted the rank-order stability of Need for Cognition with that of established personality and motivational constructs. The rank-order stability of Need for Cognition between Grade 7 and Grade 11 assessments was estimated at *r* = 0.50. This value was slightly lower than earlier estimates obtained among children and late adolescents (Bergold et al. 2023; Bergold and Steinmayr 2023), possibly because we tracked our respondents over a longer period of time, which is known to reduce rank-order stability (Bleidorn et al. 2022). The rank-order stability of Need for Cognition was comparable to that of the Big Five traits observed in the same sample and well above the rank-order stability of academic intrinsic motivation.

This finding emphasizes that Need for Cognition, as the desire to engage in thinking, has to be conceptually clearly distinguished from motivation for school. For youth, school is an important provider of cognitive activities, and Need for Cognition and academic motivation are thus quite strongly correlated (Gottfried et al. 2017; Lavrijsen et al. 2023). However, this study suggests that Need for Cognition is less malleable than academic motivation. A possible conceptualization of the difference between Need for Cognition and academic intrinsic motivation might be that Need for Cognition represents a more stable personality characteristic that drives children towards cognitive activities in general; whether this also translates into high academic motivation then depends on the school learning environment. In particular, it has been shown that high Need for Cognition is associated with increased intrinsic school motivation, particularly when teachers succeed in providing their students with appropriately challenging schoolwork (Lavrijsen et al. 2021): students with high Need for Cognition find most pleasure and fulfillment in their schoolwork when they are challenged to invest a high degree of effort in their school tasks. In contrast, when tasks are fairly easy, Need for Cognition, as the preference for cognitive challenge, is less of an asset. Of note, contemporary models on the development of individual abilities into achievement, such as the Mega Model of Talent Development (Subotnik et al. 2012) and the Talent Development in Achievement Domains (TAD) framework (Preckel et al. 2020), highlight that the attainment of a certain level of expertise depends not only on the possession of intellectual abilities but also on individual personality preferences and a set of psychosocial skills. Specifically, in the TAD framework, Need for Cognition is often cited as a key individual attribute driving children and adolescents to approach complex tasks and allocate more cognitive resources to master such tasks. In this regard, Need for Cognition might be the driver that incentivizes children to strongly commit to developing their cognitive talents in school (i.e., academic motivation). However, more research is needed to investigate the interplay between Need for Cognition and academic motivation.

### 4.2. Interindividual Variability in the Development of Need for Cognition

Second, we investigated potential interindividual variability in the intraindividual development of Need for Cognition over time. In line with earlier studies (Bergold et al. 2023; Bergold and Steinmayr 2023), we observed a significant random time slope of Need for Cognition across adolescence, although the statistical significance of slope variances became marginal when the quadratic term was added.

Latent class growth models suggested three distinct subgroups of trajectories over time. The majority of adolescents belonged to a trajectory class with medium and relatively stable levels of Need for Cognition. About one fifth of the sample reported high initial levels of Need for Cognition, which slightly decreased across adolescence, while another fifth reported low but slightly increasing levels of Need for Cognition. Hence, whereas there were small differences between subgroups in the direction of Need for Cognition over time (stable, decreasing, increasing), the subgroups were mainly differentiated by the initial level of Need for Cognition, with none of the subgroups ‘catching up’ with another subgroup throughout adolescence. This evokes the question of where these differences in ‘initial’ level—that is, in early adolescence—originate from: Do they reflect more or less innate constellations (e.g., genetic predispositions)? Are they the result of processes that affected earlier development (e.g., experiences as a child) and which mostly continue throughout adolescence? Indeed, it might be that adolescence is already a late stage in the development of Need for Cognition, with most variability occurring when children are younger. Future research could study Need for Cognition over an even longer period of time (e.g., throughout childhood and early adulthood) or adopt genetically informed research designs (Bates and Lewis 2012) to shed light on this question.

### 4.3. Individual and Environmental Factors Affecting the Development of Need for Cognition

Finally, we investigated which factors explained changes in Need for Cognition over time, that is, which factors explained class membership of Need for Cognition trajectories.

First, we found that cognitive ability was the main driver of class membership, having a moderate positive effect on belonging to the high Need for Cognition class (relative to the medium Need for Cognition class) and a moderate negative effect on belonging to the low Need for Cognition class. This is in line with intellectual investment theory, which posits that cognitive ability would affect the cognitive experiences of children by determining their probability of achieve success in cognitive efforts (i.e., environmental success hypothesis; Ackerman 1996; Ziegler et al. 2012). As children with high cognitive ability would be more successful in their cognitive activities, they would adopt a more positive attitude towards such efforts, whereas children with lower cognitive abilities would rather start to depreciate cognitive activities that might be more frustrating to them (Jebb et al. 2016). Reciprocally, high Need for Cognition would lead children to invest more in learning activities, strengthening their cognitive abilities in turn (i.e., environmental enrichment hypothesis). Of note, as explained above, our Need for Cognition subgroups were differentiated mainly by their initial levels rather than by their development over time; in the high Need for Cognition subgroup, the direction of the development across adolescence was even slightly downward, whereas in the low Need for Cognition subgroup, it was slightly upward. Hence, in line with another study among late adolescents (Bergold et al. 2023), high cognitive ability seemed to be mainly related to high initial levels of Need for Cognition, rather than to a strong shifts over time.

Second, this study addressed how the home environment affected children’s Need for Cognition. Theoretically, it has been suggested that Need for Cognition could be fostered by frequently exposing children to enjoyable and rewarding cognitive stimuli at home (see Aerts et al. 2024, regarding this issue). Because parents in socially advantaged environments often expose their children to a larger array of cognitive activities at home than socially disadvantaged parents (Rosen et al. 2020), it has sometimes been proposed that high SES children would report higher Need for Cognition. However, to date, empirical studies found little support for this thesis (Bergold et al. 2023; Colling et al. 2022; Padgett et al. 2010; Steinmayr et al. 2023). The present study further scrutinized this relationship between family environment and child Need for Cognition by investigating associations of Need for Cognition both with a distal indicator of children’s cognitive home environment, that is, parental educational level, and with a more proximal indicator, that is, the extent to which children are cognitively stimulated at home.

Bivariately, we did observe a positive association between parental educational level and child Need for Cognition, but even this bivariate association was only weak (*r* = 0.12). Moreover, when cognitive stimulation at home was controlled for, the parental educational level did not predict child membership of either the low or high Need for Cognition trajectory class. This suggests that the association between the distal variable parental educational level and Need for Cognition is mediated by cognitive stimulation at home as a more proximal variable. This finding resonates with findings from research on academic motivation, which has also reported that social background affected school motivation indirectly, that is, through cognitive stimulation, but did not have any direct effects on academic motivation (Gottfried et al. 1998).

A key finding in our study was that cognitive stimulation at home did positively predict membership of the high Need for Cognition class and negatively predict membership of the low Need for Cognition class. Hence, when parents cognitively stimulate their children, these experiences might culminate over time into a more enduring positive disposition towards thinking. Given the many academic (Liu and Nesbit 2023), motivational (Lavrijsen et al. 2021), and long-term life-course benefits (Cacioppo et al. 1996) of holding a positive disposition towards cognitive effort, it thus may be essential for parents to consider how they might act and behave to provide such a home environment to their children. Arguably, just ensuring that children are provided a number cognitive activities would not be sufficient. Rather, it matters as well whether these activities are truly stimulating children and whether children experience their parents as supportive of their cognitive activity. For example, when parents force cognitive activities on their children, children might risk experiencing these activities as pressuring and demanding and start to prefer to avoid voluntary engagement in such activities (Leslie 2014). In particular, we found that when parents acknowledged and respected their children’s views and preferences (i.e., when they supported their child’s autonomy), this led children to adopt a more positive stance towards cognitive activities. In the domain of academic motivation, parental autonomy support has been repeatedly found to be a key ingredient of improving students’ autonomous motivation for school, that is, studying because of enjoyment and interest (Soenens and Vansteenkiste 2005; Vasquez et al. 2016). In contrast, when children experience their parents as pressuring, even a high degree of parental involvement seemed to backfire, with children disengaging from their schoolwork. The current study suggests that a similar line of thought could be applied to Need for Cognition, and that the benefits of autonomy-supportive parenting could be extended from school motivation to a broader and more enduring positive disposition towards cognitive activity. Further research could further unravel what other aspects of cognitive stimulation affect Need for Cognition in children, such as the frequency of cognitive activities, their alignment with children’s interests, the degree of perceived enjoyment by children, the level of challenge, and the experience of support from parents, peers, and teachers.

Finally, we concluded by reiterating that, all in all, stability in Need for Cognition across adolescence was relatively high, and that trajectory classes were mainly differentiated by initial values rather than by different developmental directions. An important avenue for further research might be to develop and test more focused interventions aimed at improving Need for Cognition in youth. Investigating the effects of such interventions would be very informative because they might increase the variability in both the predictors and Need for Cognition in the study sample (i.e., when part of the sample receives the intervention and can be compared to a control group). For example, cognitive ability and Need for Cognition arguably evolve rather gradually over time. Hence, it might be difficult to clearly observe their reciprocal reinforcement by following a community sample in their natural environment, in which neither of both factors changes dramatically (Bergold et al. 2023). In contrast, an experimental study in which children were provided with cognitive training succeeded in eliciting both a short-term increase in cognitive abilities and an increase in Need for Cognition in the children in the treatment group (Ackermann et al. 2022). Given that the current study suggests that the home environment might play a key role in Need for Cognition development, a highly promising avenue for further research would be to develop interventions aimed at improving cognitive stimulation by parents (or other important actors in the cognitive environment of children, such as teachers) and to test whether such programs could indeed improve Need for Cognition in children.

### 4.4. Strengths, Limitations, and Directions for Future Research

This study had considerable strengths. First, it was the first longitudinal study to track adolescents’ Need for Cognition over an extended time frame across all of adolescence (i.e., between Grades 7 and 12). Second, the stability of Need for Cognition was contrasted to those of personality traits and academic intrinsic motivation, assessed within the same sample. Third, an array of individual and environmental factors theoretically assumed to affect Need for Cognition was measured, allowing us to investigate how these factors were related to the development of Need for Cognition.

Some limitations of this study have to be acknowledged. First, we only collected information on adolescents’ Need for Cognition in the first two grades and the last two grades of secondary education (i.e., Grades 7, 8, 11 and 12). As we did not collect information on Need for Cognition in Grades 9 and 10, our analyses could not address in detail how Need for Cognition developed in between Grades 8 and 11. Hence, it might be possible that shorter-term patterns in Need for Cognition development remained undetected (e.g., a sharp peak or drop in Need for Cognition in Grade 9 or 10). Further research could document the development of Need for Cognition across adolescence using more (e.g., in each grade) and/or more equally spaced measurements. Similarly, as we compared the long-term rank-order stability of Need for Cognition, personality traits, and academic intrinsic motivation between Grades 7 and 11, this study did not investigate short-term rank-order stability (e.g., 1-year stability in either young or old adolescents).

Second, the environmental predictors of Need for Cognition were restricted to the home environment. Adolescents may also engage with cognitive effort in other environments, particularly at school. Hence, factors in the school environment (e.g., how teachers provide cognitive challenge in their classes) could thus also affect Need for Cognition development, in particular in adolescents. Future research could thus also take the school environment into account and investigate how cognitive stimulation and autonomy support at school would affect Need for Cognition.

Finally, a drawback of long-term longitudinal studies is that there is often considerable attrition of respondents (Barry 2005). Also, in this study, a significant share of adolescents dropped out over the course of the study. Moreover, on average, students dropping out between Grades 7 and 8 and between Grades 8 and 11 had lower initial Need for Cognition than those who remained in this study. Dropout was primarily related to schools discontinuing their participation after Grade 8: as the study initially targeted only Grades 7 and 8, participation in later waves was optional for schools. However, school-average initial Need for Cognition was higher in schools that continued their participation throughout this study relative to schools discontinuing their participation after Grade 8. It may have been that schools with many high Need for Cognition students felt more inclined to keep participating in this study, for example, because they sympathized more with this study’s focus. In addition, Flemish secondary education is tracked, with schools often offering courses from a single track. Students changing from one track to another (which usually means changing to a less academically demanding track) thus may have been forced to leave this study. To the extent that high Need for Cognition students would have been less inclined to change to an academically less demanding track, this could further explain why the initial Need for Cognition of study dropouts was lower than for those who remained in this study. Of note, explicit individual decisions to discontinue participation (when the school as a whole kept participating) were very rare; as students completed the surveys during school hours, students may have seemed to be naturally inclined to keep participating in the surveys. All in all, the considerable degree of dropout and the (albeit small) relationship between dropout and initial levels of Need for Cognition call for some caution in interpreting results from this study; future research could do well to try to minimize student attrition, for example, by putting in effort to individually administer surveys to students who have left a participating school (e.g., in their new school or at home).

## 5. Conclusions

In a large longitudinal sample of 3409 adolescents from Flanders, the latent rank-order stability of Need for Cognition between Grades 7 and 11 was observed to equal *r* = 0.50, which was of similar magnitude to those of the Big Five personality traits and larger than that of academic intrinsic motivation. Trajectories of Need for Cognition over time were found to vary between adolescents, and a low, a medium, and a high Need for Cognition trajectory could be distinguished. Cognitive ability, cognitive stimulation at home, and parental autonomy support, but not parental educational level, were found to predict membership of these trajectories in Need for Cognition over time.

## Figures and Tables

**Table 1:** Descriptive Statistics: Means, Standard Deviations, and Correlations between Manifest Study Variables at their First Measurement Occasion.

Measure		*M*	*SD*	(1)	(2)	(3)	(4)	(5)	(6)	(7)	(8)	(9)	(10)
(1)	Need for Cognition	3.03	0.79										
(2)	BF—Openness	4.83	0.95	0.21 *									
(3)	BF—Conscientiousness	4.58	1.14	0.17 *	0.24 *								
(4)	BF—Extraversion	4.74	1.13	0.00	0.09 *	−0.09 *							
(5)	BF—Agreeableness	5.59	0.73	0.17 *	0.41 *	0.38 *	0.14 *						
(6)	BF—Neuroticism	3.80	1.16	0.01	0.08 *	0.06 *	−0.42 *	0.03					
(7)	Academic intrinsic motivation	2.77	0.86	0.40 *	0.17 *	0.28 *	0.01	0.23 *	0.02				
(8)	Cognitive ability	104.3	14.10	0.24 *	0.10 *	−0.07 *	−0.02	−0.06 *	−0.03	−0.08 *			
(9)	Parental educational level	2.99	0.83	0.12 *	0.06 *	−0.03	0.02	−0.01	−0.04	0.02	0.28 *		
(10)	Cognitive stimulation at home	2.77	0.47	0.17 *	0.11 *	0.05 *	0.00	0.07 *	0.02	0.16 *	0.01	0.17 *	
(11)	Parental autonomy support	4.10	0.59	0.11 *	0.03	0.11 *	0.02	0.04	−0.03	0.06 *	0.14 *	0.03	0.08 *

Note. * *p* < 0.01. BF: Big Five.

**Table 2:** (**a**) The results of measurement invariance tests for Need for Cognition, the Big Five traits and academic intrinsic motivation between Grades 7 and 11. (**b**) The results of measurement invariance tests for Need for Cognition between Grades 7, 8, 11 and 12.

(**a**)						
**Model**	**CFI**	**ΔCFI**	**RMSEA**	**ΔRMSEA**	**SRMR**	**ΔSRMR**
Configural	0.952	-	0.043	-	0.030	-
Metric	0.947	−0.005	0.045	0.002	0.031	0.001
Scalar	0.936	−0.011	0.047	0.002	0.034	0.003
(**b**)						
**Model**	**CFI**	**ΔCFI**	**RMSEA**	**ΔRMSEA**	**SRMR**	**ΔSRMR**
Configural	0.998	-	0.017	-	0.016	-
Metric	0.998	−0.000	0.019	0.002	0.028	0.012
Scalar	0.992	−0.006	0.032	0.013	0.033	0.005

**Table 3:** The latent correlations of Need for Cognition, the Big Five traits and academic intrinsic motivation between Grades 7 and 11.

	*r*	SE
Need for Cognition	0.50	0.02
Openness	0.67	0.03
Conscientiousness	0.55	0.02
Extraversion	0.52	0.03
Agreeableness	0.33	0.03
Neuroticism	0.48	0.03
Academic Intrinsic Motivation	0.38	0.03

**Table 4:** The latent growth curves in Need for Cognition over Grades 7, 8, 11 and 12.

	Model Fit		Parameter Estimates				
	BIC	ΔBIC		Est.	*p*	σ²	*p*
Intercept only	18,120.126		Intercept	2.975	<.001	0.328	<.001
Intercept + Linear	17,987.630	−132.50	Intercept	2.998	<.001	0.429	<.001
			Linear	−0.012	.005	0.012	<.001
Intercept + Linear + Quadratic	17,969.343	−18.29	Intercept	3.023	<.001	0.440	<.001
			Linear	−0.105	<.001	0.056	.083
			Quadratic	0.020	<.001	0.002	.222

**Table 5:** The model fits of latent class solutions with 1 to 5 classes.

Number of Classes	BIC	ΔBIC	Entropy
1 class	19,873.73	-	-
2 classes	18,546.01	−1327.71	0.58
3 classes	18,149.57	−396.44	0.61
4 classes	18,080.99	−68.58	0.59
5 classes	18,061.45	−19.54	0.48

**Table 6:** The size and growth parameter estimates for three trajectory classes of Need for Cognition.

	*N*	Share	Intercept		Linear Growth		Quadratic Growth	
			Est.	*p*	Est.	*p*	Est.	*p*
Class 1: “Medium NFC”	2347	61.4%	3.048	<.001	−0.107	<.001	0.019	<.001
Class 2: “High NFC”	699	18.3%	3.950	<.001	−0.026	.550	−0.007	.445
Class 3: “Low NFC”	778	20.3%	2.159	<.001	−0.135	.005	0.040	<.001

Note. NFC = Need for Cognition.

**Table 7:** The results of a multinomial regression model predicting the probability of belonging to either the high or low Need for Cognition trajectory class, relative to the medium class.

	**Predicting Membership of High Need for Cognition Class**				
	**β**	**SE**	**β/SE**	***p***	**Odds Ratio**
Intercept	−7.25	0.64	−11.29	-	-
Cognitive ability	0.45	0.06	8.07	<.001	1.57
Parental educational level	0.04	0.06	0.69	.488	1.04
Cognitive stimulation at home	0.24	0.05	4.51	<.001	1.28
Parental autonomy support	0.15	0.06	2.66	.008	1.16
	**Predicting membership of Low Need for Cognition class**				
	**β**	**SE**	**β/SE**	***p***	**Odds Ratio**
Intercept	3.46	0.57	6.05	-	-
Cognitive ability	−0.37	0.06	−6.16	<.001	0.69
Parental educational level	−0.08	0.05	−1.57	.117	0.92
Cognitive stimulation at home	−0.24	0.05	−4.45	<.001	0.79
Parental autonomy support	−0.04	0.05	−0.77	.438	0.96

## Data Availability

Data are unavailable due to privacy and ethical restrictions.

## Note

1 It has been noted that α’s above 0.90 do not only underscore the measurement precision of the scale but may also reflect redundancy in the items (Streiner 2003). The Need for Cognition scale has been constructed to mirror the underlying theoretical framework by Mussel (2013), in which a general factor and two specific factors nested under the general NFC factor (i.e., approaching intellectually challenging situations and expending effort to master these challenges) were proposed. For more information on the structural analysis and psychometric properties of the scale, see Keller et al. (2016) and Lavrijsen et al. (2023).

## Appendix A

**Table A1:** Correlations between manifest study variables across all measurement occasions.

		*M*	*SD*	(1)	(2)	(3)	(4)	(5)	(6)	(7)	(8)	(9)	(10)	(11)	(12)	(13)	(14)	(15)	(16)	(17)	(18)	(19)
(1)	Need for Cognition—G7	3.02	0.80																			
(2)	Need for Cognition—G8	2.98	0.82	0.60																		
(3)	Need for Cognition—G11	2.97	0.72	0.47	0.51																	
(4)	Need for Cognition—G12	3.09	0.71	0.38	0.40	0.60																
(5)	BF—Openness—G7	4.78	0.98	0.23	0.15	0.15	0.17															
(6)	BF—Openness—G11	4.38	1.00	0.15	0.19	0.31	0.20	0.43														
(7)	BF—Conscientiousness—G7	4.55	1.16	0.17	0.12	0.01	0.02	0.25	0.03													
(8)	BF—Conscientiousness—G11	4.23	1.29	0.07	0.09	0.15	0.09	0.06	0.11	0.49												
(9)	BF—Extraversion—G7	4.75	1.11	−0.00	−0.01	0.02	0.03	0.04	0.05	−0.10	−0.04											
(10)	BF—Extraversion—G11	4.44	1.33	0.03	0.01	0.01	0.05	0.09	0.10	0.02	−0.06	0.45										
(11)	BF—Agreeableness—G7	5.57	0.77	0.18	0.11	0.06	0.06	0.42	0.12	0.38	0.16	0.10	0.10									
(12)	BF—Agreeableness—G11	5.47	0.85	0.09	0.12	0.14	0.13	0.12	0.33	0.21	0.33	0.03	0.18	0.25								
(13)	BF—Neuroticism—G7	3.78	1.18	0.02	0.03	−0.00	−0.02	0.13	0.03	0.08	0.01	−0.42	−0.18	0.07	−0.00							
(14)	BF—Neuroticism—G11	3.97	1.35	−0.08	−0.04	−0.08	−0.07	0.04	0.04	−0.00	0.06	−0.16	−0.42	0.08	0.03	0.40						
(15)	Academic Intrinsic Motivation—G7	2.76	0.87	0.40	0.31	0.18	0.15	0.18	0.06	0.29	0.10	−0.01	0.03	0.23	0.13	0.03	−0.02					
(16)	Academic Intrinsic Motivation–G11	2.39	0.83	0.24	0.28	0.50	0.32	0.09	0.25	0.05	0.25	−0.02	−0.00	0.07	0.17	0.06	0.00	0.31				
(17)	Cognitive Ability	102.8	14.30	0.22	0.22	0.24	0.15	0.10	0.07	−0.06	−0.13	−0.02	−0.07	−0.03	−0.10	−0.02	−0.09	−0.07	−0.02			
(18)	Parental Educational Level	2.96	0.84	0.11	0.11	0.10	0.07	0.06	0.05	−0.02	−0.07	0.01	0.04	−0.00	−0.02	−0.03	−0.05	0.01	0.07	0.28		
(19)	Cognitive Stimulation at Home	2.78	0.48	0.16	0.12	0.15	0.12	0.11	0.11	0.04	0.00	−0.00	0.04	0.06	0.05	0.01	−0.04	0.15	0.15	0.01	0.17	
(20)	Parental Autonomy Support	4.09	0.59	0.11	0.09	0.05	0.10	0.03	−0.00	0.10	0.09	0.01	0.03	0.04	−0.01	−0.03	−0.00	0.05	0.04	0.14	0.05	0.08

Note. BF: Big Five.
